# Interest of the thoracic scanner in the diagnosis of COVID-19: study of 35 cases in the Republic of Guinea

**DOI:** 10.11604/pamj.supp.2020.35.133.24549

**Published:** 2020-08-05

**Authors:** Hugues Ghislain Atakla, Kaba Condé, Mahugnon Maurel Ulrich Dénis Noudohounsi, Malcom Steves Sokeng Dongmo, Aichatou Hamidou Garba, Dismand Stephan Houinato, Fodé Abass Cissé

**Affiliations:** 1 Neurology Department, Ignace Deen University Hospital Center, Conakry, Guinea,; 2 Public Health Physician, Research Project Manager, Brazzaville, Congo,; 3 Pneumology Department, Ignace Deen University Hospital Center, Conakry, Guinea,; 4 Neurology Department, Hubert Koutoukou Maga University Hospital Center, Cotonou, Benin

**Keywords:** COVID-19, RT-PCR, chest CT, Guinea

## Abstract

**Introduction:**

the aim of this work is to evaluate the contribution of thoracic computed tomography (CT) in the diagnosis of COVID-19 in Guinea.

**Methods:**

this was a retrospective study with data recorded over a 2 Month period. Records of patients who tested positive on chest CT without contrast injection on admission were included in this study. Not included are those who did or did not perform a chest CT scan after confirmation of the diagnosis by RT-PCR. The data were collected under the direction of the National Health Security Agency (ANSS) and analysed using STATA/SE version 11.2 software.

**Results:**

all patients tested performed a chest CT scan without contrast injection while awaiting the RT-PCR test result. Eighty percent (80%) of patients had lesions characteristic of COVID-19 viral pneumonia on chest CT. The reverse transcriptase PCR (RT-PCR) test was later positive in 33 patients (94.28%) and negative in 2 (5.71%).

**Conclusion:**

it is noted from this study that chest computed tomography is a critical tool in the rapid diagnosis of COVID-19 infection. Its systematization in all patients suspected in our dispute, would facilitate diagnosis while waiting for confirmation by RT-PCR and would limit the loss of cases.

## Introduction

A series of pneumonias caused by coronavirus 2019 (SARS-CoV-2) was reported in Wuhan, China in late 2019 with unprecedented expansion in the rest of the world. The World Health Organization (WHO) declared the coronavirus epidemic a pandemic on 11 March 2020 [1]. To date, there are 7,495,776 cases worldwide [2] with 4258 positive cases recorded to date in Guinea Conakry [3]. In contrast to Severe Acute Respiratory Syndrome (SARS) and Middle Eastern Respiratory Syndrome (MRS), asymptomatic patients with COVID-19 have been reported to be contagious [4]. COVID-19's transmission capacity may be greater than that of SARS. SARS CoV-2 can be easily transmitted from person to person through close contact, droplets and aerosols [5,6]. The most common symptoms of VCOS-RAS-2 infection include fever; cough; myalgia; physical asthenia; and less common symptoms such as productive cough; hemoptysis; and diarrhea [7,8]. The incubation period is 5.1 days [9]. Thus, early diagnosis and immediate isolation of positive patients are crucial to break the chain of transmission and thus control the epidemic. To date, the polymerase chain reaction retro-transcriptase (RT-PCR) is the Gold Standard for the diagnosis of COVID-19. However, there is a high rate of false negatives, and a shortage of RT-PCR, resulting in delayed diagnosis and loss of cases in our context. Apart from the clinical presentation, chest lesions visible on a chest CT scan are essential for early diagnosis and evaluation of the disease [10] as they allow COVID-19-induced pneumonia to be quickly distinguished from other viral pneumonias. The typical CT involvement of COVID-19 pneumonia consists of bilateral, peripheral subpleural, often posterior and basal frosted glass areas [11]. In this study, we analyzed 35 patients with symptoms suspicious of COVID-19 and diagnosed positive on chest CT despite negative RT-PCR test. The aim of the work is to expose the advantage of thoracic computed tomography (CT) in the diagnosis of COVID-19 in order to establish its systematization in front of any suspicion of SARS-Cov-2 in Guinea.

## Methods

It was a retrospective study carried out over a period of 2 months from 30 March to 30 May 2020. It involved all patients (3415) presenting symptoms of SARS-Cov-2 infection and followed up at the national centre for the fight against infectious pathologies in Conakry. Only those (35) who performed a chest CT scan without injection of contrast material while awaiting the result of the RT-PCR test were included. Not included are those who did or did not perform a chest CT scan after confirmation of the diagnosis by RT-PCR. The clinical data analyzed were age, sex, COVID-19 infectivity, comorbidity, symptoms, and chest CT results. All images were consistently analyzed by a certified respirologist and chest radiologist. Patient data were collected and analyzed using STATA/SE version 11.2 software. Anonymity without implying any potential risk to patients and in compliance with the ethical rules in agreement with the National Health Security Agency (ANSS) was the norm. There was no connection between patients and researchers.

## Results

**Figure 1:**
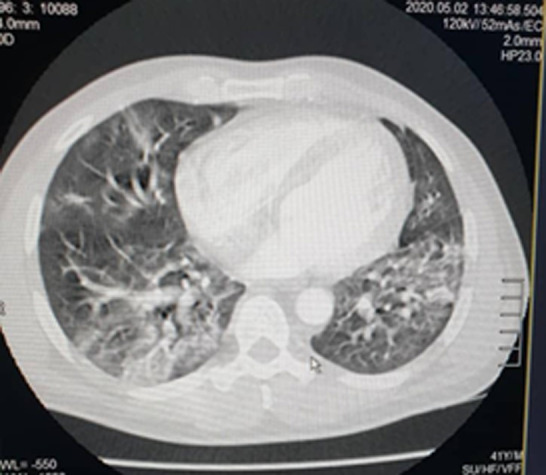
posterior peripheral and basal lesions

**Figure 2:**
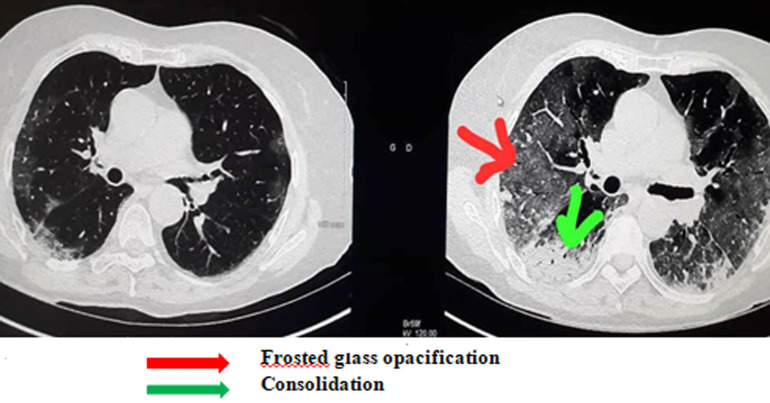
frosted glass opacification consolidation

**Figure 3:**
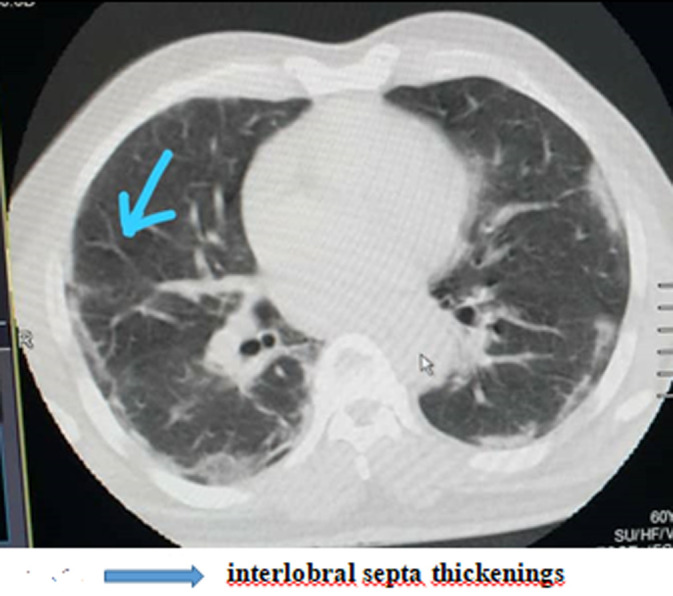
interlobral septa thickenings

Of the 35 patients followed in this study, 23 were male with an M/F sex ratio of 1.92. The mean age of the patients was 41 ± 9.034 years. The anamnesis revealed that 22.85% of the patients had been in contact with a patient confirmed positive for COVID-19, compared to 77.14% who were unaware of having been in contact or not with a person carrying the virus. Co-morbidities of cardiovascular disease and smoking were found in 20% of patients. The predominant symptoms in the clinic were respectively: flu-like symptoms; fever above 39°C and dyspnea distributed in the following proportions: 94.28%; 88.57; 54.28. Other clinical characteristics of patients are summarized in (Table 1). Due to the delay in RT-PCR diagnosis in our setting, the patients in this study were those who performed a chest CT scan without contrast injection while waiting for the RT-PCR result. Eighty percent (80%) of patients had lesions characteristic of COVID-19 viral pneumonia on chest CT. The reverse transcriptase PCR (RT-PCR) test was later found to be positive in 33 patients (94.28%) and negative in 2 patients (5.71%) whose CT scan was positive for COVID-19. The CT scan showed a sensitivity of 80% versus a specificity of 0% (Table 2). Lesions were peripheral, posterior and basal in 65.71% of cases (Figure 1); and 80% were multifocal and bilateral. Of the 35 patients who tested negative for RT-PCR, 28 patients or 80% had characteristic lesions with peripheral, multifocal and bilateral frosted glass opacification type and consolidation in a proportion of 22.85% (Figure 2). Multifocal and bilateral presence of Kerley lines (thickening of the interlobar septa) was noted in 54.28% of patients (Figure 3). In addition, the degree of severity of the lesions was assessed by the radiologist in percentage terms, taking into account the volume of the pulmonary parenchyma. It was noted that 28.57% of the lesions were minor (lesion < 25% of parenchymal volume); moderate lesions were 25-50% and only 2.85% of the lesions were greater than 50% of parenchymal volume (Table 3).

**Table 1:** distribution of patients according to clinical data

Features: Clinic /Paraclinics		Staff (n)	Percentage (%)
**Clinic**	**Average age (year)**		**41 ans**
	Men	23	65,71
	Women	12	34,28
**Exposure history**	Notion of patient contact covid-19	8	22,85
	Unknown exposure	27	77,14
**Comorbid**	Cardiovascular disease	7	20
	Others	16	45,71
	Smoking vice	7	20
**Signs and symptoms**	Fever	31	88,57
	Dry cough	16	45,71
	influenza-like illness	33	94,28
	Dyspnea	19	54,28
	Sore throat	6	17,14
	Digestive signs	11	31,42
	Myalgia	9	25,71
	Other symptoms	3	8,57

**Table 2:** distribution of 35 patients by RT-PCR and CT scan results

		RT-PCR		
		Positive	Negative	Total
**Chest CT Scan**	Positive	26	2	28
	Negative	7	0	7
	Total	33	2	35

**Table 3:** patient distribution by paraclinical outcomes

		Staff (n)	Percentage (%)
**Nucleic acid test by Real-time PCR**	Positive	33	94,28
	Negative	2	5,71
	RT-PCR negative and CT positive	2	5,71
**Normal chest CT scan**		7	20
**Location of lesions**	Posterior peripheral and basal lesions	23	65,71
	Unilateral injury	0	
	Bilateral multifocal lesions	28	80
**Number of lobes involved**	None	7	20
	Only one lobe	0	
	More than two lobes involved	28	80
**Characteristics of the lesions**	Frosted glass opacification	28	80
	Kerley Line	19	54,28
	Consolidation	8	22,85
**Degree of severity according to parenchymal lesions**	Lesion < 25%	10	28,57
	Lesion [25%-50%]	17	48,57
	Lesion ˃ 50%	1	2,85

## Discussion

According to the currently available diagnostic criteria of COVID-19, the nucleic acid test is the main diagnostic tool for COVID-19 [12]. However, the test result is very time-consuming and may even give false negative results due to laboratory error or insufficient viral material in the specimen [13]. Among the 3415 patients followed up at the national centre for infectious diseases, 35 patients underwent chest CT scan, the result of which was characteristic of a COVID-19 viral infection in 80% (28/35) of cases. This result is comparable to that of Ai *et al*. who reported in 88% (888/1014) of initially negative patients, scan characteristics suggesting the presence of COVID-19 [14]. The RT-PCR test was later positive in 33 patients (94.28%) and negative in 2 patients (5.71%) whose CT scan was positive for COVID-19. These various reports show that in some cases, patients may have negative RT-PCR results from nasopharyngeal or throat swabs and at the same time present CT features typical of the disease. Many authors have already reported similar results. Bingjie Li et al. found false negatives with a positive CT scan on a symptomatology suggestive of COVID-19 [10]. It is believed that the conditions under which the examination is performed and the sample is stored are conducive to the negativity of the test in our context. Both sexes were found with a sex ratio of 1.91. The average age was 41 ± 9.034 years. More than half (77.14%) of the patients are unaware of having been in contact with people carrying the virus in their daily activities. Since the disease is contagious even to asymptomatic patients [15], the risk of transmission and spread is always considerable. This probably explains why 27 (77.14%) of the patients presented in this work felt that they did not know whether they had been in contact with subjects carrying the virus. The clinical symptoms found in this series are consistent with those reported in the literature [7,8].

Eighty percent (80%) of patients had lesions characteristic of COVID-19 viral pneumonia on chest CT. Coronavirus 2019 disease has different imaging manifestations at different stages. Early stage COVID-19 lesions are relatively localized and are manifested primarily by inflammatory infiltration limited to the subpleural or peribronchovascular regions of one or both lungs. We report in this work, 20% negative chest CT. Very few cases present negative CT scan results at an early stage [16]. The sensitivity of the chest CT to covivid-19 is already known [16]. It should be noted that the chest CT scan had a sensitivity of 80% versus a specificity of 0% in this work. This means that there is an 80% chance that a patient diagnosed positive for COVID-19 on chest CT will be confirmed positive by RT-PCR. However, there are many abnormalities to look for on the chest CT scan of patients suspected of being COVID-19 positive. Extensive ground glass opacities, peripheral multifocal and bilateral opacities were found in all of our CT positive patients. According to some recent studies [11,17], lesions classically predominate in the peripheral, posterior and basal regions. The results of this study show a peripheral redistribution of the lesions with more marked involvement of the posterior and basal areas in 65.71% of patients. Pulmonary consolidation was found as an associated lesion in 22.85% of patients. This feature of CT scanning in patients with COVID-19 is worrisome as it is considered a sign of disease progression [18]. According to some reports, greater consolidation indicated disease progression, while absorption and smaller size of these lesions indicated improvement [19,20]. In addition, thickening of the interlobar septa in 54.28% of patients, indicating an interstitial syndrome. Taking into account the parenchymal volume, lesions less than 25% are considered minor and those between 25-50% are said to be moderate. This result illustrates the extent of the lesions caused by VCOS-RAS-2 on the lung and justifies the respiratory distress which is most often symptomatologically consistent. Treatment was symptomatic in all patients and the outcome was favourable in all. We believe that a future study on monitoring the evolution of CT lesions will provide a better understanding of the pathogenesis in order to limit damage to the lungs.

## Conclusion

Although the positive nucleic acid test remains the reference examination for the positive diagnosis of COVID-19, the characteristics of the scanner can be used for the clinical diagnosis of infection despite negative nucleic acid test results. This study revealed lesions characteristic of COVID-19 viral pneumonia on chest CT in 80% of patients who tested negative for RT-CPR. Given the sensitivity of chest CT to COVID-19 lesions, and the irreplaceable role it has played in the detection of these patients, we believe that its systematization in all patients suspected of having COVID-19 would facilitate diagnosis and monitoring of response to treatment. It should be noted, however, that a negative chest CT scan does not rule out a diagnosis of COVID-19 infection, particularly in the early stages of the disease.

### What is known about this topic

- The reverse transcription polymerase chain reaction (RT-PCR) assay allows healthcare workers to confirm COVID-19 infection.

### What this study adds

- Thoracic CT scan can be used as a diagnostic tool for COVID-19 infection in case of false negatives or in case of lack of RT-PCR.
